# Reversal Effects of 20(R)- and 20(S)-Ginsenoside-Rg3 on Daunorubicin Uptake in Multidrug-Resistant Leukemia Cells Studied in the Single-Cell Biochip

**DOI:** 10.3390/ijms27062661

**Published:** 2026-03-14

**Authors:** Yuchun Chen, Nandini Joshi, Megan Chiem, Iryna Kolesnyk, Paul C. H. Li, Patrick Y. K. Yue, Ricky N. S. Wong

**Affiliations:** 1 Department of Chemistry, Simon Fraser University, Burnaby, BC V5A 1S6, Canada; 2 Department of Biology, Hong Kong Baptist University, Hong Kong, China

**Keywords:** microfluidic biochip, single cell fluorescence, leukemia drug uptake, multidrug resistance, daunorubicin efflux reversal, ginsenosides

## Abstract

Multidrug resistance (MDR), frequently mediated by overexpression of the P-glycoprotein (P-gp) efflux transporter, remains a major challenge in the treatment of leukemia by limiting intracellular accumulation of chemotherapeutic agents such as daunorubicin (DNR). This study evaluates the applicability of a microfluidic-based single-cell biochip to investigate the reversal effects of microgram-level ginsenosides on daunorubicin uptake in multidrug-resistant leukemia cells. Pure ginsenosides are difficult to obtain in bulk and are typically available only in milligram quantities, which restricts their evaluation using conventional MDR assays such as flow cytometry that require large cell populations and substantial amounts of compounds. To address this limitation, a microfluidic single-cell biochip (SCB) requiring microgram quantities of ginsenosides (<100 µg) and fewer than ten cells was employed. Intracellular DNR accumulation was measured in the CEM/VLB1000 leukemia cell line following treatment with DNR alone or in combination with ginsenoside Rg3-R, ginsenoside Rg3-S, 20(S)-protopanaxatriol (PPT), and 20(S)-protopanaxadiol (PPD), in order to compare their relative efficacy in enhancing drug accumulation. Although Rg3-R and Rg3-S share highly similar chemical structures and are glycosylated derivatives of the PPD aglycone, Rg3-S exhibited greater potency in increasing intracellular daunorubicin accumulation than Rg3-R, and both were more effective than PPD. These findings underscore the importance of ginsenoside stereochemistry modulating P-gp-associated drug resistance and demonstrate the utility of the SCB platform for quantifying daunorubicin accumulation in multidrug-resistant leukemia cells at single-cell resolution.

## 1. Introduction

Multidrug resistance (MDR) remains a major obstacle to effective cancer chemotherapy, posing a substantial challenge to disease modulation and long-term treatment success. One of the most extensively studied mechanisms underlying MDR is the overexpression of the ATP-binding cassette (ABC) transporter called P-glycoprotein (P-gp/ABCB1), which actively effluxes a wide range of chemotherapeutic agents from cancer cells, thereby reducing intracellular drug accumulation and therapeutic efficacy [1,2,3,4]. In leukemia, elevated P-gp expression has been strongly associated with the resistance to anthracycline drugs such as daunorubicin (DNR), often leading to treatment failure and disease relapse [5].

We have previously reported a same-single-cell approach for studying the modulation of drug efflux in multidrug-resistant cancer cells using a microfluidic platform [6]. This approach was later extended to evaluate drug modulation by directly measuring intracellular drug accumulation rather than efflux [7]. Compared with the earlier efflux-based method, the accumulation-based approach is both faster and simpler while maintaining single-cell resolution. This methodology has since been adopted as the microfluidic single-cell biochip (SCB) platform for subsequent MDR studies [8].

In parallel with advances in microfluidic technologies, increasing attention has been directed toward traditional medicinal compounds due to their reported anticancer properties and potential to enhance chemotherapy outcomes, including improved therapeutic response and enhanced quality of life [9]. Among these compounds, ginsenosides, bioactive saponins derived from Panax ginseng, have attracted significant interest. Several studies have suggested that MDR in cancer cells can be reversed by ginsenosides, potentially through interactions with the P-gp transporter that reduce drug efflux [10,11,12]. As a result, intracellular accumulation of chemotherapeutic agents such as DNR may be enhanced in MDR cells.

Despite their therapeutic potential, purified ginsenosides are not easy to test because they are difficult to obtain in bulk and are typically available only in low amounts at the milligram level. This limitation presents a great challenge for conventional MDR assays, such as flow cytometry, which require large numbers of cells and milligram quantities of compounds in each experiment [13,14]. To overcome this constraint, the microfluidic single-cell biochip (SCB) method was developed to enable MDR evaluation using microgram quantities of compounds like ginsenosides and fewer than ten cells per experiment. In this study, the SCB platform was applied to measure intracellular DNR accumulation in single MDR leukemia cells using microgram quantities of ginsenosides (<100 µg), including Rg3.

Ginsenoside Rg3 has been reported to possess anticancer activity [15] and is present at low levels in white (air-dried) ginseng but at higher concentrations in steamed (red) ginseng [16,17,18]. Elevated levels of Rg3 have also been identified in certain commercial ginseng products [19]. There were four ginsenosides studied in this paper: 20(S)-ginsenoside Rg3 (Rg3-S), 20(R)-ginsenoside Rg3 (Rg3-R), 20(S)-protopanaxadiol (PPD) and 20(S)-protopanaxatriol (PPT). Rg3-S and Rg3-R are structurally similar as they are the two stereoisomers at the C-20 position of Rg3. In addition, PPD and PPT share similar core frameworks, with PPD serving as the aglycone of both Rg3 stereoisomers. While these compounds are all structurally similar, they exhibit distinct biological activities [20].

Moreover, only Rg3 and PPD have been reported to have the MDR-reversal effect [16,21,22]. However, differences in MDR-reversal activity between the Rg3 stereoisomers, Rg3-R and Rg3-S, have not been systematically investigated, particularly at the single-cell level. In this work, we employed the SCB platform to evaluate how four structurally related ginsenosides, Rg3-S, Rg3-R, PPD, and PPT, affect intracellular DNR accumulation in individual multidrug-resistant leukemia cells. This study aims to clarify the influence of ginsenoside stereochemistry on MDR modulation while further demonstrating the applicability of microfluidic single-cell analysis for drug resistance studies using scarce natural products.

## 2. Results and Discussion

### 2.1. MDR-Reversal Effect on DNR Accumulation in the MDR Cancer Cells

**Figure 1:**
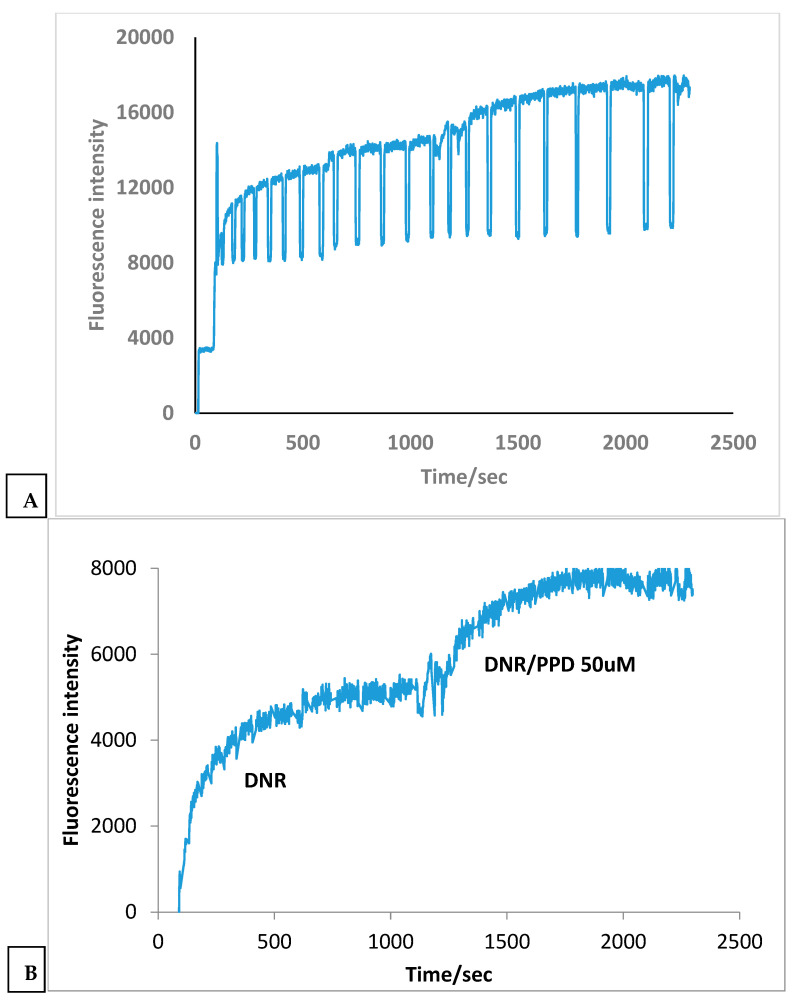
Uptake enhancement of DNR due to PPD (50 µM) on a single-cell. (**A**) Raw time-resolved intracellular DNR fluorescence. (**B**) Corrected DNR accumulation trace showing enhanced intracellular DNR uptake after PPD addition.

We first evaluated the effect of PPD on intracellular DNR accumulation in MDR cancer cells. As shown in Figure 1A, when only DNR solution was given to the cell, cell fluorescence increased, indicating continuous DNR accumulation. After ~700 s, fluorescence reached a steady state, reflecting equilibrium between DNR influx and efflux, with the latter process via P-gp transporters. Upon addition of DNR solution containing Rg3-R, intracellular DNR accumulation increased, indicating MDR reversal. The DNR accumulation increased 1.6-fold in the presence of PPD, demonstrating MDR reversal (Figure 1B).

We next applied the same uptake procedure to Rg3-R, Rg3-S, and PPT. The results of the uptakes are summarized. As shown in Table 1, the ginsenosides have a dose-dependent effect on CEM/VLB1000 cells. Intracellular DNR accumulation doubled with rising ginsenoside concentrations, indicating a dose-dependent effect. This result is superior to a previous study where the effective Rg3 concentration for MDR reversal as measured by the rhodamine 123 accumulation assay was observed not at 100 µM, but at a 320 µM level, though that was measured by the cytotoxicity assay as observed at a 5–40 µM level [16].

Although Rg3-R and Rg3-S share similar chemical structures, Rg3-S was more potent at modulating MDR. The more potent effect of Rg3-S was also observed in its effect on insulin secretion [23] and anti-apoptotic effect [20].

### 2.2. DNR Accumulation Control Experiments

**Figure 2:**
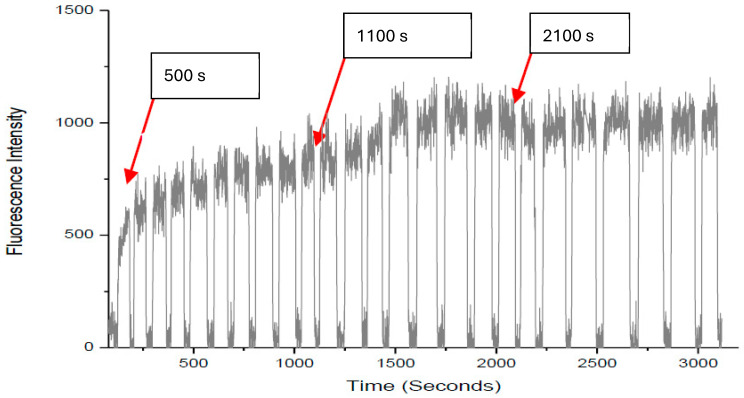
Negative control conducted in the single-cell chip.

To confirm that the increased DNR accumulation was not caused by fluctuations from solution switching, we used DNR-only solution to replace the DNR/Rg3 solution. Specifically, DNR solution was added sequentially three times to the same cell, and accumulation was measured after each addition. The rate of accumulation curve of daunorubicin 35 µM in CEM/VLB1000 subline is shown in Figure 2. DNR was added into reservoir 2 at 100 s of the drug into the cell. Subsequent to addition, internal DNR accumulation increased until 500 s, after which the accumulation reached a plateau of 940.04 and the efflux of the drug out of the cell equated to the intracellular accumulation. Furthermore, switching drugs (DNR 35 µM) at 1100 s and at 2100 s did not increase intracellular accumulation of the drug significantly.

We have repeated the same experiments on three different cells, and the results are shown in Table 2. The results clearly show that the DNR accumulation will not increase dramatically after it reaches the steady state simply by repeatedly adding DNR to the cell, thus confirming that the enhanced DNR accumulations are caused by the MDR-reversal effects of Rg3-R and Rg3-S as described in Section 2.1.

When we compared the effects of ginsenosides between two cell lines, CEM/VLB1000 and CEM/WT, it was evident that the fold increase is low in the CEM/WT, i.e., 1.70 ± 0.14 (n = 2) for 50 µM and 2.35 ± 0.50 (n = 2) for 100 µM. This may be due to the evidence that CEM/wt does not possess as many P-gp pumps as the CEM/VLB1000 subline.

## 3. Materials and Methods

Cell cultures: The drug-sensitive human leukemia CCRF-CEM cell line (CEM/WT) and the multidrug-resistant Vinblastine (VLB) subline (CEM/VLB 1000) were obtained from BC Cancer Agency. Both CEM/WT and the resistant subline, CEM/VLB 1000, were cultured in α-MEM supplemented with 10% fetal bovine serum (FBS) and penicillin (5%). The cell lines were maintained at 37 °C in a humidified atmosphere containing 5% CO_2_ and were passaged once a week. In addition, the drug-resistant subline, CEM/VLB1000, was sub-cultured with 100 µg/mL vinblastine solution to maintain resistance of 1000 µg/mL (CEM/VLB). More information is provided in the Supplementary Information.

**Figure 3:**
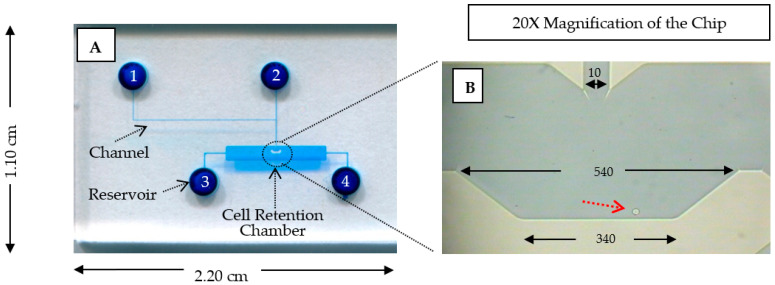
Design of the microfluidic single-cell chip. (**A**) A photograph image of the microchip colored with blue food dye. The microchip consists of 4 solution reservoirs and 1 cell-retention chamber. The dimensions of the microchip are 1.10 cm × 2.20 cm. (**B**) An image of the retained CEM/VLB1000 cell (as pointed by the red arrow) under 20× magnification, with chamber and channel dimensions shown in µm. The channel depth is 40 µm.

Microchip fabrication: Figure 3 depicts the design of the microfluidic single-cell chip, with the chip specification given in the caption. The microfluidic chip was fabricated using the standard micromachining procedures on glass by CMC Microsystems (Kingston, ON, Canada). These procedures, which include standard chip cleaning, thin metal film deposition, photolithography, photoresist development, glass etching by hydrofluoric acid, reservoir forming on cover plate, and chip bonding, have been described previously [24]. As shown in Figure 3A, the microfluidic chip consists of four reservoirs connected by four microchannels to a central cell-retention chamber. In the chamber, there is a V-shaped structure, which can retain a single cell. Reservoir 1 is the α-MEM solution inlet, reservoir 2 is the reagent inlet, reservoir 3 is the cell inlet, and reservoir 4 is the waste outlet. A microfluidic chip was prepared and cleaned with NOX soap, purified water and 90% ethanol, consecutively.

Drug accumulation: Prior to the experiment, an aliquot (~100 µL) of CEM/VLB1000 cell culture suspension was taken for each of the experiments. The microfluidic chip was mounted on the stage of an inverted fluorescence microscope, and the equipment was connected to a CCD camera interfaced with a computer. Before introducing the vial with the cells, the chip was sterilized by introducing 70% ethanol through reservoir 4 and allowing it to flow through the microchannels. After 70% ethanol was pipetted out of reservoir 4, α-MEM was added to the remaining chip reservoirs. Next, about 10 µL of the cell sample was drawn by a micropipette and added into reservoir 3 of the microfluidic chip. By manually adjusting the pressure inside the microfluidic chip, one cell was selected and retained within the central chamber for the experiment. A measurement window was set up, and the drug accumulation was monitored by repeatedly positioning the cell within a defined measurement window over a total period of 3100 s (the first 100 s were used for background measurement, after which the reagent solutions were introduced at 1000 s intervals). The accumulation curves were processed and background-corrected [5,8,25]. The fold increases were calculated by normalizing the DNR accumulation in the presence of inhibitors to the baseline DNR accumulation measured in the same single cells.

## 4. Conclusions

We evaluated the intracellular DNR accumulation in the presence of four ginsenosides: Rg3-S, Rg3-R, PPD, and PPT. Using the CEM/VLB1000 leukemia cell line, we compared the intracellular DNR accumulation in the presence of each ginsenoside to evaluate their relative efficacy at reversing MDR-mediated drug efflux. It was found that although Rg3-R and Rg3-S share similar chemical structures and are aglycones of PPD, Rg3-S exhibited greater potency in enhancing the intracellular DNR accumulation, and both Rg3 stereoisomers were more effective than PPD.

## Figures and Tables

**Table 1:** Drug accumulation fold increases in the CEM/VLB cell also treated with ginsenosides.

	DNR (35 µM) Only or with Added Compound					
	DNR 35 µM Only		DNR 35 µM + PPT	DNR 35 µM + PPD	DNR 35 µM + Rg3-S	DNR 35 µM + Rg3-R
**35 µM (A)**	1.35 ± 0.06 (n = 4)	**(B) 50 µM**	2.14 ± 0.67 (n = 5)	1.68 ± 0.15 (n = 4)	2.15 ± 0.47 (n = 4)	2.05 ± 0.24 (n = 4)
**p1**			0.06	0.02	0.04	0.01
**35 µM (2)**	1.38 ± 0.22 (n = 4)	**(C) 100 µM**	3.52 ± 1.06 (n = 5)	2.30 ± 0.36 (n = 4)	5.90 ± 1.89 (n = 4)	3.52 ± 0.98 (n = 5)
**p2**			0.04	0.03	0.03	0.05
**p**			0.004	0.0007	0.0006	0.001

*p* is the probability value of one-way ANOVA test for (A) DNR control, (B) DNR + reagent (50 µM) and (C) DNR + reagent (100 µM). p1 and p2 are the values of Student’s *t* tests; p1 is for the comparison of A with B, whereas p2 is for that of B with C. All differences are statistically significant as *p* < 0.05, except for 50 µM PPT.

**Table 2:** DNR accumulation in the MDR cancer cells untreated with any other reagents.

	DNR Accumulation		
	Cell 1	Cell 2	Cell 3
DNR 35 µM 1	1.4	1.3	1.3
DNR 35 µM 2	1.3	1.2	1.3
Cell image	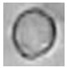	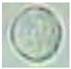	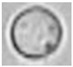

## Data Availability

The original contributions presented in this study are included in the article/Supplementary Material. Further inquiries can be directed to the corresponding author.

## Supplementary Materials

The following supporting information can be downloaded at: https://www.mdpi.com/article/10.3390/ijms27062661/s1.
