# Supplementary material for: Reversal Effects of 20(R)- and 20(S)-Ginsenoside-Rg3 on Daunorubicin Uptake in Multidrug-Resistant Leukemia Cells Studied in the Single-Cell Biochip
Source: Int J Mol Sci. 2026 Mar 14;27(6):2661. doi: 10.3390/ijms27062661 (PMC13026126; doi:10.3390/ijms27062661)
Supplement: Supplementary file 1 [file ijms-27-02661-s001.zip › ijms-4088058-supplementary.pdf]

Supplementary Information

Reversal effects of 20(R)– and 20(S)–ginsenoside-Rg3 on daunorubicin uptake in multidrug resistant leukemia cells studied in the single-cell biochip

Yuchun Chen,[1] Nandini Joshi,[1] Megan Chiem,[1] Iryna Kolesnyk,[1] Paul C.H. Li,[1*] Patrick Y.K. Yue,[2] Ricky N.S. Wong[2]

[1] Department of Chemistry, Simon Fraser University, Canada

[2] Department of Biology, Baptist University, Hong Kong

*Correspondence author

Supplementary Materials: Raw data for the uptake of drugs in the CEM-CCRF cell treated with VLB (1000 ug/ml) 1-06-30

		DNR 35 µM uptake			
		Cell 1<br>404.04	Cell 2<br>940.04	Cell 3<br>335.01	Cell 4<br>462.11
DNR 35 µM Control	35µM (1)	1.3	1.3	1.4	1.4
	35µM (2)	1.3	1.2	1.7	1.3
	Cell Morphology /Size (um)	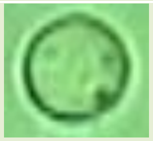<br>11	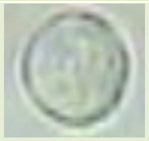<br>12	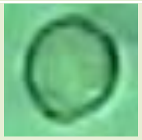<br>10	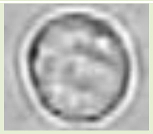<br>12
	Cell Passage number/date cultured	Passage 1<br>10-03-03	Passage 2	Passage 7	Passage 10
	Experiment Date	0-03-04	0-03-11	0-04-12	0-10-21

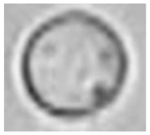

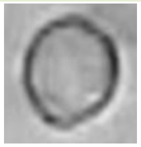

		DNR 35 µM uptake				
		Cell 1 480.55	Cell 2 277.27	Cell 3 369.78	Cell 4 820.91	Cell 5 429.90
PPT Ginsenoside Fold Enhancement	50µM	2.3	2.8	2.7	1.3	1.6
	100µM	3.6	4.1	4.8	2.0	3.1
	Cell Morphology /Size (um)	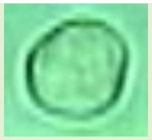 10	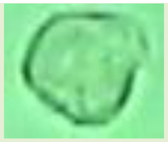 11	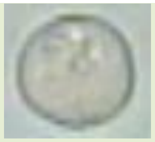 12	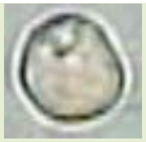 12	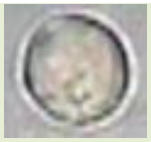 13
	Cell Passage number/date cultured	No Data	No Data	Passage 8 0-05-10	Passage 10	Passage 16 0-07-19
	Experiment Date	0-02-16	0-02-25	0-05-14	0-07-10	0-07-22

		DNR 35 µM uptake			
		**Cell 1** 604.92	**Cell 2** 563.36	**Cell 3** 582.60	**Cell 4** 335.10
PPD Ginsenoside Fold Enhancement	50µM	1.5	1.6	1.8	1.8
	100µM	2.0	2.0	2.7	2.5
	Cell Morphology /Size (um)	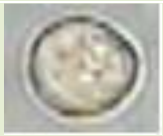 12	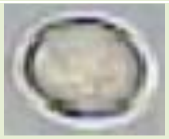 12	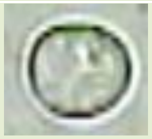 11	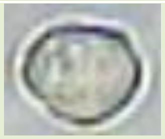 12
	Cell Passage number/date cultured	Passage 9 0-05-12	Passage 9 0-05-12	Passage 11	Passage 0 0-08-02
	Experiment Date	0-05-20	0-05-21	0-06-10	0-08-03

		Cell 1<br>291	Cell 2	Cell 3<br>2141	Cell 4<br>623	Cell 5<br>415
DNR 35 μM uptake						
Rg3-S Ginsenoside Fold Enhancement	50μM	2.8	Fiber problem	1.7	2.0	2.1
	100μM	8.5		4.0	5.3	5.8
		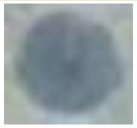<br>(Cell died)	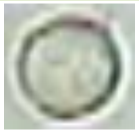<br>10	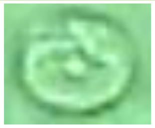<br>(Cell died)	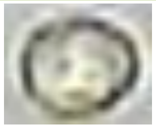<br>9	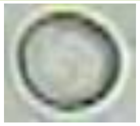<br>11
Cell Passage number/date cultured		Passage 3<br>1-06-13	Passage 3<br>1-06-13	Passage 3<br>1-06-13	Passage 3<br>1-06-13	Passage 3<br>1-06-13
Experiment Date		1-06-17	1-06-21	1-06-22	1-06-24	1-06-24

		Cell 1<br>1300	Cell 2<br>800	Cell 3<br>300	Cell 4<br>850	Cell 5<br>750
**DNR 35 μM uptake**						
Rg3-R Ginsenoside Fold Enhancement	50μM	2.0	1.9	2.4	First added DNR 35uM/Rg3R 100	1.9
	100μM	2.6	3.1	5.0	4.0	2.9
		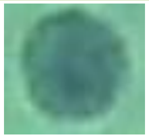<br>(Cell died)	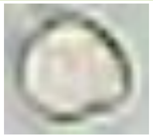<br>10	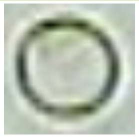<br>10	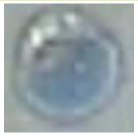<br>(Cell died)	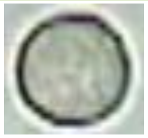<br>12
Cell Passage number/date cultured						Passage 3<br>1-06-13
Experiment Date		1-06-08	1-06-13	1-06-14	1-06-14	1-06-15

CEM wt Data

WT CELL DNR 35 µM uptake		
	**Cell 1** 436.45	**Cell 2** 710.35
50µM	1.8	1.6
100µM	2.7	2.0
Cell Morphology /Size (um)	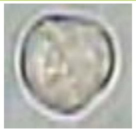 16	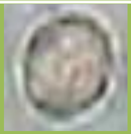 11
Cell Passage number/date cultured	Passage 16 0-07-19	Passage 3
Experiment Date	0-07-20	0-08-30

SD/Mean

Mean/SD Table for the Ginsenosides and VLB Cell – Based on the data above

		Compound					
		DNR 35 uM		PPT/ DNR 35uM	PPD/ DNR 35uM	Rg3-S/ DNR 35uM	Rg3-R/ DNR35uM
Concentration	[35] uM (1)	1.35 ± 0.06 (n=4)	[50] uM	2.14 ± 0.67 (n=5)	1.68 ± 0.15 (n=4)	2.15 ± 0.47 (n=4)	2.05 ± 0.24 (n=4)
	[35] uM (2)	1.38 ± 0.22 (n=4)	[100]uM	3.52 ± 1.06 (n=5)	2.30 ± 0.36 (n=4)	5.90 ± 1.89 (n=4)	3.52 ± 0.98 (n=5)

Mean/SD Table for the Ginsenosides and WT Cell – Based on the data above

		Compound	
			**PPT/ DNR 35uM**
**Concentration**		**[50] uM**	1.70 ± 0.14 (n=2)
		**[100]uM**	2.35 ± 0.50 (n=2)

Supplementary Materials: Data for Table 1 and ANOVA

DNR	+PPT50	+PPT100	DNR	+PPD50	+PPD100	DNR	Rg3-S50	Rg3-S100	DNR	Rg3-R50	Rg3-R100
1.3	2.3	3.6	1.3	1.5	2	1.3	2.8	8.5	1.3	2	2.6
1.3	2.8	4.1	1.3	1.6	2	1.3	1.7	4	1.3	1.9	3.1
1.4	2.7	4.8	1.4	1.8	2.7	1.4	2	5.3	1.4	2.4	5
1.4	1.3	2	1.4	1.8	2.5	1.4	2.1	5.8	1.4	1.9	4
	1.6	3.1									2.9
p-value		0.003735			0.000668			0.000631			0.001283

Anova: Single Factor PPT

SUMMARY

Groups	*Count*	*Sum*	*Average*	*Variance*
Column 1	4	5.4	1.35	0.003
Column 2	5	10.7	2.14	0.443
Column 3	5	17.6	3.52	1.117

ANOVA

Source of Variation	*SS*	*df*	*MS*	*F*	*P-value*	*F crit*
Between Groups	11.02	2	5.51	9.697	0.003735	4
Within Groups	6.25	11	0.568			
Total	17.27	13				

Anova: Single Factor PPD

SUMMARY

Groups	*Count*	*Sum*	*Average*	*Variance*
Column 1	4	5.4	1.35	0.003
Column 2	4	6.7	1.675	0.023
Column 3	4	9.2	2.3	0.127

ANOVA

Source of Variation	*SS*	*df*	*MS*	*F*	*P-value*	*F crit*
Between Groups	1.865	2	0.933	18.34	0.000668	4.3
Within Groups	0.458	9	0.051			
Total	2.323	11				

Anova: Single Factor Rg3S

SUMMARY

Groups	*Count*	*Sum*	*Average*	*Variance*
Column 1	4	5.4	1.35	0.003
Column 2	4	8.6	2.15	0.217
Column 3	4	23.6	5.9	3.58

ANOVA

Source of Variation	*SS*	*df*	*MS*	*F*	*P-value*	*F crit*
Between Groups	47.21	2	23.6	18.63	0.000631	4.3
Within Groups	11.4	9	1.267			
Total	58.61	11				

Anova: Single Factor Rg3R

SUMMARY

Groups	*Count*	*Sum*	*Average*	*Variance*
Column 1	4	5.4	1.35	0.003
Column 2	4	8.2	2.05	0.057
Column 3	5	17.6	3.52	0.957

ANOVA

Source of Variation	*SS*	*df*	*MS*	*F*	*P-value*	*F crit*
Between Groups	11.17	2	5.586	13.94	0.001283	4.1
Within Groups	4.008	10	0.401			
Total	15.18	12				
